# Combined effects of 17-DMAG and TNF on cells through a mechanism related to the NF-kappaB pathway

**DOI:** 10.1186/1746-1596-8-70

**Published:** 2013-05-01

**Authors:** Zhuling Qu, He Dong, Xiaolin Xu, Wei Feng, Xuanlong Yi

**Affiliations:** 1 The Affiliated Hospital of Medical College, Qingdao University, Qingdao, Shandong province 266021, China

**Keywords:** 17-DMAG, IKKβ, TNF, NF-κB Pathway

## Abstract

**Objective:**

The tumor necrosis factor (TNF) and the cellular NF-κB pathway protein IKKβ play important roles in various cellular processes such as cell proliferation, survival, differentiation, and apoptosis. A heat shock protein 90 inhibitor, 17-DMAG, can induce apoptosis of some tumor cells. This study is to determine the combined effects of 17-DMAG and TNF on malignant cells and the related mechanisms.

**Methods:**

We have determined effects of 17-DMAG, an Hsp90 inhibitor, and TNF treatments on the small cell lung cancer cell line (MS-1), the adenocarcinoma cell line (A549), the squamous-cell carcinoma cell line (LK-2), and the normal human bronchial epithelium cell line (NuLi-1) by using the 3-(4,5-dimethylthiazol-2-yl)-2,5-diphenyltetrozolium bromide assay. To determine if 17-DMAG inhibit the expression of IKKβ in the normal human NuLi-1 cells, and the malignant MS-1, A549, and LK-2 cells, immunoblotting assays and luciferase assays were performed.

**Results:**

It was found that the combined treatments resulted in synergistic killing of malignant cells, which was confirmed by the apoptosis determination using a fluorescence microscopic assay following staining of the drug-treated cells with Hoescht 33258. The immunoblotting results indicated that the synergistic killing due to 17-DMAG and TNF treatments may be related to the decreases in IKKβ levels in the presence of 17-DMAG.

**Conclusions:**

The results suggest that combination of 17-DMAG and TNF treatments might be useful for treating malignancies upon further study in the further.

**Virtual slides:**

The virtual slide(s) for this article can be found here: http://www.diagnosticpathology.diagnomx.eu/vs/2041198513886824

## Introduction

Heat shock proteins (Hsps) are a group of chaperones that are important in maintaining stability and function of their client proteins. Heat shock protein 90 (Hsp90) regulates stability, degradation, translation, and function of its client proteins, via an ATP-regulated mechanism [1]. Unlike other Hsps, only approximately 200 cellular proteins are determined to be clients of Hsp90 [2-4]. Hsp90 inhibitors, such as 17-DMAG, are a group of small molecules that bind to the ATP-binding pocket of the Hsp90 dimers [5,6]. By competing with ATP, 17-DMAG can inhibit the protein chaperoning activity of Hsp90, resulting in misfolding of cellular client proteins such as the tyrosine kinase v-Src, the serine/threonine kinase Raf, mutant p53 proteins, the glucocorticoid receptor, and the androgen receptor HER2, resulting in protein destabilization and degradation, usually mediated by the proteasomal pathway [7]. A number of Hsp90 clients are involved in tumor developments [7]. In addition, the binding affinity of Hsp90 inhibitors to the ATP-binding pocket of Hsp90 are 10-flod higher in tumor cells than in normal cells [8]. Therefore, Hsp90 inhibitors are promising therapeutic reagent for treating malignant cells.

The tumor necrosis factor (TNF) plays important roles in various cellular processes such as cell proliferation, survival, differentiation, and apoptosis [9]. Since TNF can induce apoptosis of cells, it may be a potential therapeutic agent to treat cells. However, TNF induces multiple pathways including cell proliferation and survival processes that may facilitate tumor development [10]. Therefore, combination of TNF and another agent will be a promising method of treating malignant cells, if this agent can down-regulate the cell proliferation property of TNF. Therefore, novel reagents are necessary to inhibit TNF-induced survival signals, possibly by resulting in a synergistic effect on malignant cells.

When TNF binds to the TNF receptors, a group of signaling pathways were activated. For example, the caspase cascade can be activated through the Fas-associated death domain protein, which leads to apoptosis [10]. However, activation of the NF-κB pathway through the receptor-interaction protein upon binding of TNF facilitates cell survival and proliferation [11-13], which will decrease the anti-tumor apoptotic properties of TNF. Therefore, the balance of TNF-induced survival and death signaling is essential for the fates of cells in the presence of TNF.

During TNF-induced signaling, IκB kinases (IKKs), including IKKα, IKKβ, and IKKγ, are recruited to the signaling complex. The activated IKKβ then phosphorylates the NF-κB-bound IκBs, which retain NF-κB in the cytoplasm, at their regulatory region to trigger their degradation. This process releases NF-κB to allow its translocation into the nucleus and activation of its target genes [14,15], with many of them possessing anti-apoptotic properties [16-18]. Since IKKβ has been identified as an Hsp90 client protein [19,20], we hypothesize that combined treatments with 17-DMAG and TNF may lead to a promising anti-tumor effects if 17-DMAG can decrease the survival signaling properties of TNF.

Since TNF and 17-DMAG may be useful for treating some types of malignancies. In this study, we have determined the effects of 17-DMAG and TNF treatments on multiple cell lines. It is found that the combined treatments result in synergistic killing of cells. It is also indicated that such a synergistic killing may be related to the decreases in IKKβ levels in the presence of 17-DMAG. Our results suggest that combination of 17-DMAG and TNF might be useful for treating malignancies upon further study in the further.

## Materials and methods

### Cell lines and reagents

The small cell lung cancer (SCLC) cell line (MS-1), the adenocarcinoma cell line (A549), the squamous-cell carcinoma cell line (LK-2), and the normal human bronchial epithelium cell line (NuLi-1) were provided by Shanghai Cell Biology Institute (China). These lung cancer cell lines were maintained in RPMI-1640 medium (Sigma-Aldrich Co. Ltd, Irvine, CA) supplemented with 10% fetal bovine serum (FBS), 1% L-glutamine, and 1% penicillin/streptomycin at 37°C with 5% CO_2_ and 100% humidity. 17-DMAG (InvivoGen, San Diego, CA) and TNF-α (TNF) were dissolved in DMSO. Recombinant Human TNF was purchased from Promega.

### Cell treatments and the 3-(4,5-dimethylthiazol-2-yl)-2,5-diphenyltetrozolium bromide (MTT) assay

Briefly, cells at a density of 1 × 10^5^ cells/well were seeded into 6-well plates in RPMI-1640 supplemented with 10% calf serum and were cultured for 24 h. The cells were then treated with vehicle control (DMSO, 0.016%, v/v), TNF-α (10 ng/ml), 17-DMAG (0.05 μM), or TNF-α (10 ng/ml) plus 17-DMAG (0.05 μM). At the end of each experiment, cells were incubated with 0.5 mg/ml MTT for 4 h according to the protocol of manufacturer. Viability of treated cells was expressed relative to control cells (relative viability).

### Apoptosis assay

Cells at a density of 1 × 10^5^ cells/well were cultured in six-well plates in RPMI-1640 supplemented with 10% calf serum for 24 h, followed by addition of DMSO (0.016%, v/v), TNF (10 ng/ml), 17-DMAG (0.05 μM), or TNF (10 ng/ml) plus 17-DMAG (0.05 μM). After 48 h, cells were pelleted by centrifugation, washed once with PBS, fixed by incubation in 4% paraformaldehyde for 30 min at room temperature, and then washed again with PBS to remove the fixative. The fixed cells were resuspended in PBS that contained Hoescht 33258 (5 μg/ml), followed by an incubation at room temperature for 15 min in the dark. Aliquots of cells were placed on glass slides and examined for cells with apoptotic morphology (nuclear condensation and chromatin fragmentation) via fluorescence microscopy. To quantify the apoptosis, 300 nuclei from random microscopic fields were analyzed. Data are presented as the mean percentages of apoptotic cells.

### Immunoblotting assays

Cells at a density of 1 × 10^5^ cells/well were cultured in six-well plates in RPMI-1640 supplemented with 10% calf serum for 24 h, followed by addition of DMSO (0.016%, v/v), TNF (10 ng/ml), 17-DMAG (0.05 μM), or TNF (10 ng/ml) plus 17-DMAG (0.05 μM). After 48 h, cells were pelleted by centrifugation, washed twice with PBS. Total proteins were harvested from cells, separated on 10% SDS/PAGE gels, and then subjected to immunoblot analyses. The primary antibodies against IKKβ (about 90 kDa) and β-actin were purchased from Santa Cruz, USA (anti- IKKβ, cat# sc-8014, 1:200; anti-β-actin, cat# sc-130301, 1:10,000). Secondary antibodies used in this study were goat anti-mouse IgG-HRP (Cat# sc-2005, 1:10,000, Santa Cruz, USA). Bound antibodies were detected using the ECL system (Pierce Biotechnology). The immunoblot experiments were repeated at least 3 times. The mean normalized optical density (OD) of HDAC protein bands relative to the OD of β-actin band from the same individual was calculated.

### Luciferase assays

NuLi-1 cells, MS-1 cells, A549 cells, and LK-2 cells were plated onto 24-well plates and incubated for 20 h at 37°C in 5% CO2. The control pGL3 vector DNA (0.2 μg) (purchased from Clontech Co.) or similar amounts of Luciferase-NF-κB reporter vectors (Clontech Co., USA), was cotransfected into cells with the pRL-SV40 vector using the effectene transfection reagent (Qiagen, USA) for 20 h. Cells were then treated with DMSO (0.016%, v/v), TNF (10 ng/ml), 17-DMAG (0.05 μM), or TNF (10 ng/ml) plus 17-DMAG (0.05 μM) for 24 h. Luciferase activity was measured using the Dual-Luciferase Reporter Assay System (Promega, USA) according to the manufacturer's protocol. The transcriptional activity of NF-κB in the cells transfected with the Luciferase-NF-κB reporter vectors were calculated as folds of those in the relative cells transfected with the empty vector.

### Statistical analyses

The experimental data are expressed as mean ± SD. Statistical software (SPSS10.0) was used for independent sample *t* tests, followed by one-way variance analysis. In all analyses, P < 0.05 was considered statistically significant.

## Results

### TNF-α enhances the toxic effects on tumor cells of 17-DMAG

A normal human bronchial epithelium cell line (NuLi-1) and three lung cancer cell lines (MS-1, A549, and LK-2) were treated with TNF, 17-DMAG, or both of them together for 24 hour or 48 hours. The treatment with DMSO served as a drug vehicle control. The cells were analyzed for differences in cell killing upon various treatments via number counting of living cells in the presence or absence of the above compounds.

**Figure 1:**
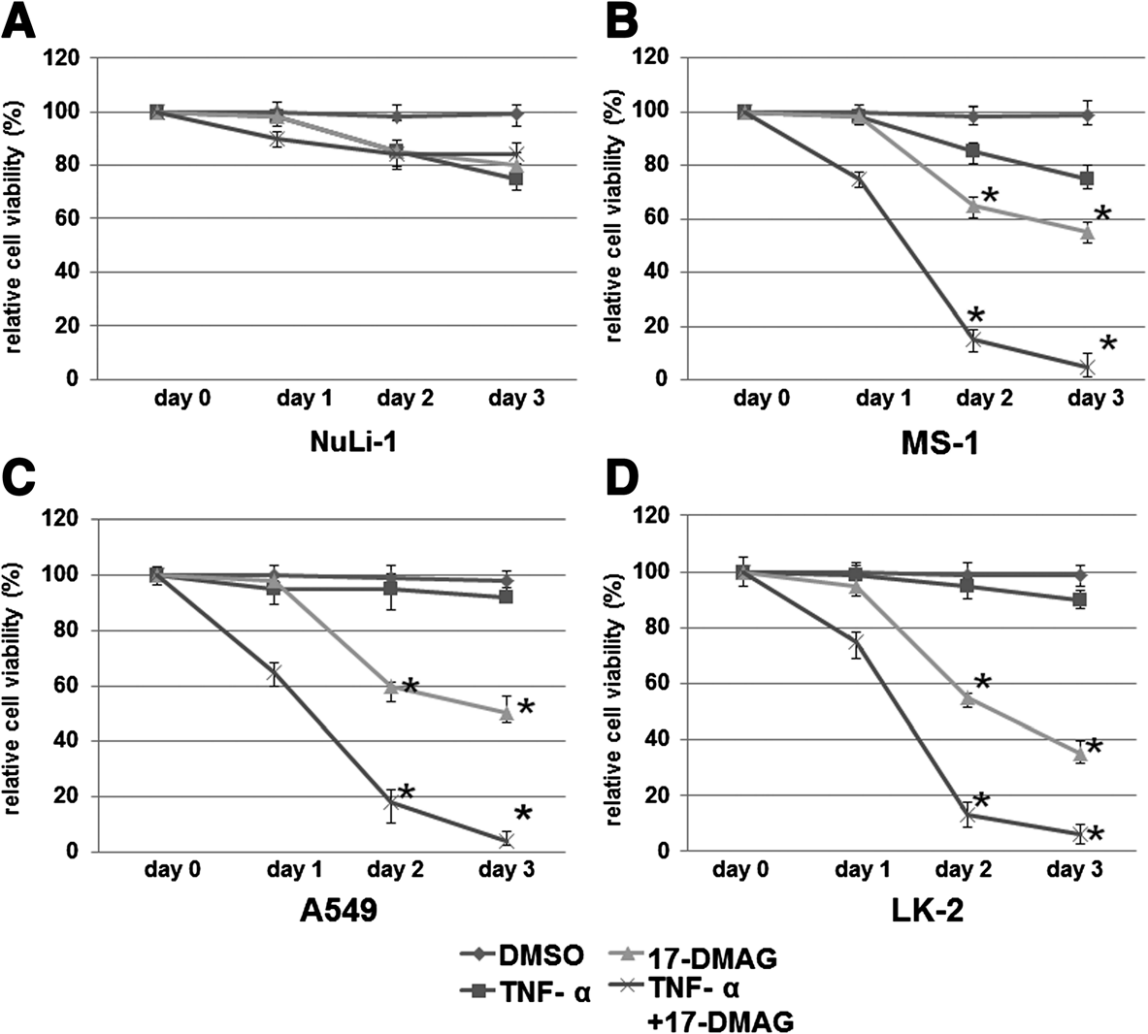
**Cell treatments with DMSO, TNF-α, 17-DMAG, or TNF-α and 17-DMAG together.** The normal human bronchial epithelium cell line (NuLi-1) and three lung cancer cell lines (MS-1, A549, and LK-2) were treated with either vehicle control (DMSO), TNF-α (10 ng/ml), 17-DMAG (0.05 μM), or both. Cell counts in each condition were determined by trypan blue exclusion at the time points indicated. (**A**) NuLi-1 cells; (**B**) MS-1 cells; (**C**) A549 cells; (**D**) LK-2 cells. Cell viability was measured using 3-(4,5-dimethylthiazol-2-yl)-2,5-diphenyltetrozolium bromide (MTT) assay immediately before (day 0) and after 1, 2, or 3 days of incubation with the drugs. Values are means ± SD for three experiments. It is considered not significant, when P > 0.05 vs. control (DMSO) cell viability of each treatment. *, it is considered as a significant difference, when P < 0.05 vs. corresponding control.

Results showed that the treatments with the drug vehicle control (DMSO) did not significantly affect cell viability of all of these four types of cells, including the normal human bronchial epithelium cell line (NuLi-1, Figure 1A) and three lung cancer cell lines MS-1 (Figure 1B), A549 (Figure 1C), and LK-2 (Figure 1D). Treatments with TNF-α had slight effects, if any, on cell viability of all of these four types of cells (Figure 1A-D). Treatments with 17-DMAG decreased viabilities of the three lung cancer cell lines MS-1 (Figure 1B), A549 (Figure 1C), and LK-2 (Figure 1D) by approximately 40% at day 2 and up to 60% at day 3, but no obvious decreases for the normal human bronchial epithelium cell line (NuLi-1, Figure 1A). When treated with TNF-α and 17-DMAG together, the viabilities of the three lung cancer cell lines MS-1 (Figure 1B), A549 (Figure 1C), and LK-2 (Figure 1D) were reduced by more than 80% at day 2 and by 90% at day 3. Combined treatments with TNF-α and 17-DMAG had not significantly decreased the viability of the normal cells (NuLi-1, Figure 1A), suggesting that such dosages of TNF-α and 17-DMAG are not toxic to normal cells. The above results suggest that TNF-α enhances the toxic effects on tumor cells of 17-DMAG.

### TNF-α enhances the apoptosis induced by 17-DMAG

Since TNF-α enhances the toxic effects on tumor cells of 17-DMAG, it was determined that the effects of the drugs on apoptosis in all of these 4 types of cells. The cells were treated with either vehicle control (DMSO), TNF-α (10 ng/ml), 17-DMAG (0.05 μM), or both of TNF-α (10 ng/ml) and 17-DMAG (0.05 μM). To quantify the apoptotic incidence, we used a fluorescence microscopic assay following staining of the drug-treated cells with Hoescht 33258.

**Figure 2:**
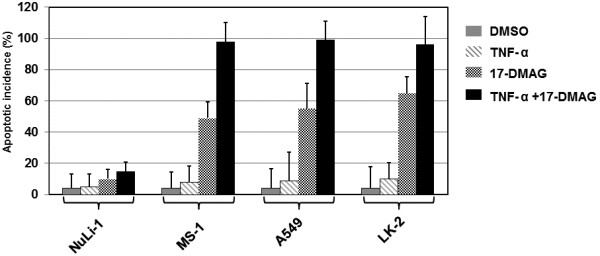
**Detection of phenotype**-**dependent apoptosis induced by treatments with DMSO, TNF**-**α, 17**-**DMAG, or TNF-α plus 17-DMAG.** The normal human bronchial epithelium cell line (NuLi-1) and three lung cancer cell lines (MS-1, A549, and LK-2) were treated with either vehicle control (DMSO), TNF-α (10 ng/ml), 17-DMAG (0.05 μM), or both of TNF-α (10 ng/ml) and 17-DMAG. Cells were harvested 48 h later. Hoechst 33258-stained cells were examined for apoptotic characteristics (nuclear margination and chromatin condensation) using a fluorescence microscope. Apoptotic incidence was calculated. Data were expressed as means ± SD for three independent experiments.

As shown in Figure 2, treatment with DMSO or TNF-α resulted in only slightly increased effects on apoptosis of all of these four types of cells. 17-DMAG caused apoptosis of MS-1, A549, and LK-2 cells with the incidences between 50-60%, although it did not alter the apoptotic incidence of the normal NuLi-1 cells significantly when compared with the DMSO treatment. It is worthy to note that the presence of TNF-α increased the 17-DAMG induced apoptosis, with the incidences up to 90% in comparison with the treatments with 17-DMAG alone. These results indicated that TNF significantly elevated the apoptosis induced by 17-DAMG, although it alone did not result in a significant induction of apoptosis.

### 17-DMAG treatments lead to degradation of IKKβ

IKKβ expression is reported to be reduced by Hsp90 inhibitors in some cell types. To determine if 17-DMAG inhibit the expression of IKKβ in NuLi-1, MS-1, A549, and LK-2 cells, the cells were treated with either vehicle control (DMSO), TNF-α (10 ng/ml), 17-DMAG (0.05 μM), or both of TNF-α (10 ng/ml) and 17-DMAG (0.05 μM). The total proteins were extracted and the expression levels of IKKβ were determined by using immunoblot analysis, with the cellular β-actin protein serving as a loading control. The mean normalized OD of IKKβ protein bands relative to the OD of β-actin band from the same condition was all calculated and subjected to statistical analyses.

**Figure 3:**
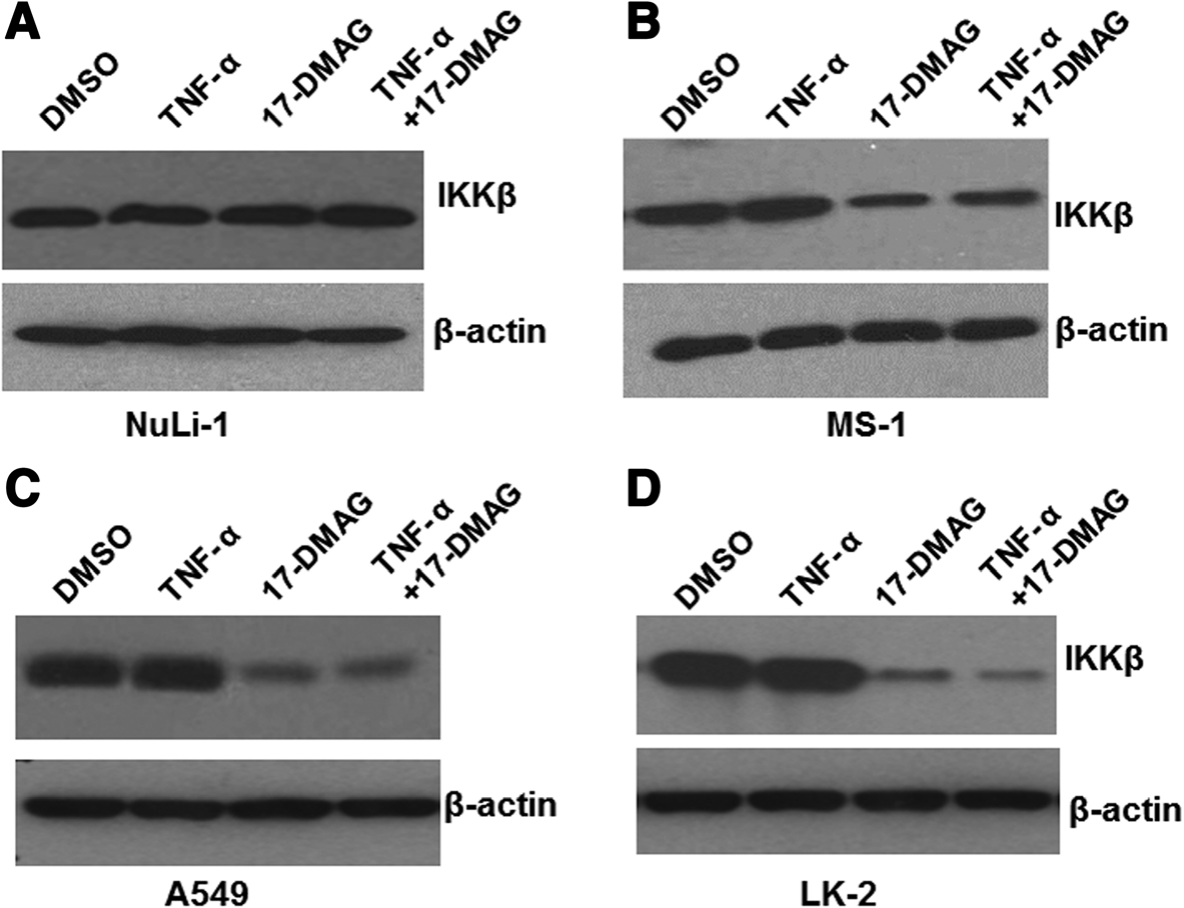
**17-DMAG decreases expression of IKKβ.** Cells were treated with DMSO, TNF-α (10 ng/ml), 17-DMAG (0.05 μM), or both of TNF-α (10 ng/ml) and 17-DMAG for 40 hours. Whole-cell extracts were prepared and immunoblot analysis was performed to analyze the expression of IKKβ, β-actin. The cellular β-actin served as a loading control. (**A**) NuLi-1 cells; (**B**) MS-1 cells; (**C**) A549 cells; (**D**) LK-2 cells.

Representative blots were shown in Figure 3. As shown in Figure 3, treatment with DMSO or TNF-α did not detectably affect IKKβ expression in all of these four types of cells. 17-DMAG, in the absence or presence of TNF-α, did not result in decreased expression of IKKβ in the normal NuLi-1 cells. However, the treatments with 17-DAMG decreased expression of IKKβ by up to 50%, 80%, and 90% in MS-1, A549, and LK-2 cells cells, respectively, according to the calculated OD values of the IKKβ bands relative to the β-actin bands. These results indicated that 17-DMAG significantly decreased IKKβ expression, although TNF-α did not affect such effect of 17-DMAG.

### 17-DMAG down-regulates the NF-κB transcriptional activity induced by TNF treatment

Next, we performed a luciferase reporter gene assay to determine whether TNF-α induces transcriptional activity of NF-κB and, if so, whether 17-DMAG affects the induction of NF-κB transcriptional activity by TNF-α. NuLi-1 cells, MS-1 cells, A549 cells, and LK-2 cells were transfected with a control pGL3 vector DNA or similar amounts of Luciferase-NF-κB reporter vectors together with the pRL-SV40 vector for 20 h.

**Figure 4:**
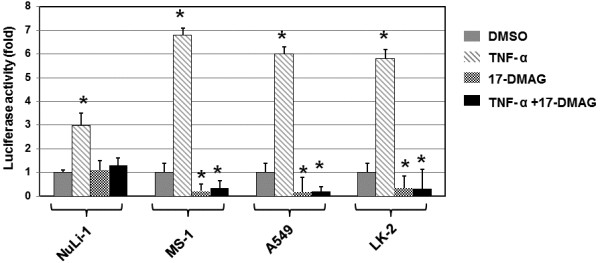
**Luciferase reporter gene assay.** NuLi-1 cells, MS-1 cells, A549 cells, and LK-2 cells were plated onto 24-well plates and incubated for 20 h at 37°C in 5% CO2. The control pGL3 vector DNA (0.2 μg) or similar amounts of Luciferase-NF-κB reporter vectors, was cotransfected into cells with the pRL-SV40 vector using the effectene transfection reagent. Cells were then treated with DMSO (0.016%, v/v), TNF (10 ng/ml), 17-DMAG (0.05 μM), or TNF (10 ng/ml) plus 17-DMAG (0.05 μM) for 24 h. Luciferase activity was measured in cell lysates. Each experiment was repeated thrice with similar results. *, it is considered as a significant difference, when P < 0.05 vs. corresponding control.

As shown in Figure 4, treatments with TNF-α resulted in 3-fold higher luciferase activities in NuLi-1 cells and about 6-flod higher luciferase activities in MS-1 cells, A549 cells, and LK-2 cells, when compared with the DMSO conditions. Treatments with 17-DMAG did not lead to detectable alteration of the luciferase activities in NuLi-1 cells, but reduced the luciferase activities in MS-1 cells, A549 cells, and LK-2 cells by about 70% in comparison with the DMSO conditions. Upon treatments with TNF-α and 17-DMAG together, the luciferase activities in NuLi-1 cells were not significantly changed, but the activities in MS-1 cells, A549 cells, and LK-2 cells were reduced by about 70-80%, in comparison with the DMSO conditions. These results indicated that 17-DMAG down-regulates the NF-κB transcriptional activity induced by TNF treatment.

## Discussion

Since many Hsp90 clients are important for disease development, 17-DMAG is extensively studied for possible treatments of various diseases [21-25]. In this study, we have determined the effects of 17-DMAG and TNF treatments on multiple cell lines. It is found that the combined treatments result in synergistic killing of malignant cells. It is also indicated that such a synergistic killing may be related to the decreases in IKKβ levels in the presence of 17-DMAG. Our results suggest that combination of 17-DMAG and TNF might be useful for treating malignancies upon further study in the further.

Combined treatments with TNF-α and 17-DMAG had not significantly decreased the viability of the normal NuLi-1 cells, but leads to obvious toxicity on malignant MS-1, A549, and LK-2 cells. This result may be related to the properties of Hsp90. As one of the most abundant proteins in the cytoplasm, Hsp90 constitutes approximately 1-2% of the total proteins [26], although some Hsp90 may translocate to the nucleus in response to stress and other environmental stimuli [27-30]. Under normal conditions of the normal cells, there is an abundant Hsp90 chaperone reservoir, which can buffer proteostasis against environmental stress. However, under extreme environmental conditions, such as conditions in malignant cells, the chaperone reservoir is rapidly exhausted. Therefore, effects of 17-DMAG may be increased correspondingly. In the other words, function of Hsp90 inhibitors can then affect the relationship between genotype and phenotype, and thus influencing human health, disease and evolutionary processes. This reason also explains why 17-DMAG did not affect IKKβ expression in normal cell.

Hsp90 is composed of a highly conserved amino-terminal domain, a middle domain, followed by a carboxy-terminal domain [31]. Hsp90 binds ATP in its amino-terminal domain, hydrolyzing it upon interaction with clients and co-chaperoning molecules. Structurally unrelated inhibitors of Hsp90, such as 17-DMAG, replace ATP and specifically destroy Hsp90 function [32]. The strong conservation of the ATP-binding pocket allows 17-DMAG to serve as extremely useful and specific tools for affecting HSP90 function. This may explain why TNF did not enhance the effect of 17-DMAG on IKKβ expression, while they exert a combined effect on viability of malignant MS-1, A549, and LK-2 cells, possibly by playing roles in different cellular signaling pathways or processes.

When cells are treated with TNF, NF-κB is activated through RIP-mediated activation of IκB kinase (IKK) and then released, allowing for its translocation into the nucleus and activation of its target genes [14]. Many cellular targets of NF-κB, such as Bcl-xL, cIAP-1, A20, cIAP-2, and XIAP, have anti-apoptotic functions [16-18]. Therefore, many kinds of tumor cells are resistant or tolerant to TNF-induced apoptosis, although TNF may lead to apoptosis via the FADD-caspase pathway and the TRAF2-JNKK2 pathway. The NF-κB activity is considered as a major factor of TNF resistance in cancer cells [33]. Our results clearly show that inhibition of the NF-κB pathway by 17-DMAG significantly sensitizes the TNF-treated cells to induced cell death, supporting the combined effects of 17-DMAG and TNF on cells through a mechanism related to the NF-κB pathway. In addition to the small-molecule compounds such as 17-DMAG, the siRNA strategies are often used to treat tumor cells. It is found that targeting the fascin pathway by using a specific siRNA might be a novel therapeutic strategy for curing the esophageal squamous cell carcinoma [34]. Compounds can be also used for study of gene expression. It has been reported that gene expression profile test is useful to resolve the lung squamous cancers and other cancers such as the head and neck squamous carcinoma [35].

## Competing interests

The authors declare that they have no competing interests.

## Authors’ contributions

ZQ and HD designed the experiments, analyzed the data, and wrote the manuscript. XX, WF, and XY did the experiments and analyzed the data. All authors read and approved the final manuscript.
